# Cold-induced metabolic conversion of haptophyte di- to tri-unsaturated C_37_ alkenones used as palaeothermometer molecules

**DOI:** 10.1038/s41598-018-20741-2

**Published:** 2018-02-02

**Authors:** Eri Kitamura, Tomonori Kotajima, Ken Sawada, Iwane Suzuki, Yoshihiro Shiraiwa

**Affiliations:** 1 Graduate School of Life and Environmental Sciences, University of Tsukuba, 1–1–1 Tennodai, Tsukuba, Ibaraki, 305–8572 Japan; 2 Faculty of Life and Environmental Sciences, University of Tsukuba, 1–1–1 Tennodai, Tsukuba, Ibaraki, 305–8572 Japan; 3 Department of Natural History Sciences, Faculty of Science, Hokkaido University, N10W8, Kita-ku, Sapporo, 060–0810 Japan; 4 CREST, JST, N10W8, Kita-ku, Sapporo, 060–0810 Japan; 5 CREST, JST, 1–1–1 Tennodai, Tsukuba, Ibaraki, 305–8572 Japan

## Abstract

The cosmopolitan marine haptophyte alga *Emiliania huxleyi* accumulates very long-chain (C_37_-C_40_) alkyl ketones with two to four *trans*-type carbon-carbon double bonds (alkenones). These compounds are used as biomarkers of haptophytes and as palaeothermometers for estimating sea-surface temperatures in biogeochemistry. However, the biosynthetic pathway of alkenones in algal cells remains enigmatic, although it is well known that the C_37_ tri-unsaturated alkenone (K_37:3_) becomes dominant at low temperatures, either by desaturation of K_37:2_ or by a separate pathway involving the elongation of tri-unsaturated alkenone precursors. Here, we present experimental evidence regarding K_37:3_ synthesis. Using the well-known cosmopolitan alkenone producer *E*. *huxleyi*, we labelled K_37:2_ with ^13^C by incubating cells with ^13^C-bicarbonate in the light at 25 °C under conditions of little if any K_37:3_ production. After stabilisation of the ^13^C-K_37:2_ level by depleting ^13^C-bicarbonate from the medium, the temperature was suddenly reduced to 15 °C. The ^13^C-K_37:2_ level rapidly decreased, and the ^13^C-K_37:3_ level increased, whereas the total ^13^C-K_37_ level—namely [K_37:2_ + K_37:3_]—remained constant. These ^13^C-pulse-chase-like experimental results indicate that ^13^C-K_37:2_ is converted directly to ^13^C-K_37:3_ by a desaturation reaction that is promoted by a cold signal. This clear-cut experimental evidence is indicative of the existence of a cold-signal-triggered desaturation reaction in alkenone biosynthesis.

## Introduction

*Emiliania huxleyi* (Division, Haptophyta) is a coccolithophore covered with coccoliths made of calcium carbonate crystals. It is widely distributed across the polar and equatorial oceans^1,2^. This unicellular calcifying phytoplankton produces huge blooms in early to mid-summer across wide areas of the ocean. It functions as a biological pump to transport CO_2_ from the surface to sediments and is therefore thought to contribute greatly to the global carbon cycle^3–5^. Recently, the growth of coccolithophore blooms in the ocean has attracted interest in the study of global climate-change effects such as global warming and ocean acidification caused by increasing atmospheric CO_2_ concentrations^6–8^.

Some coccolithophores, including *E*. *huxleyi*, produce very-long-chain neutral lipids with ketone moieties, known as alkenones^9,10^. Alkenones are either methyl (Me)- or ethyl (Et)-ketones with a C_37_–C_40_ linear acyl group containing two to four *trans*-type unsaturated bonds. Previous studies have identified a total of 17 different alkenone species from both marine sediments and laboratory-cultured coccolithophores^11,12^. Alkenones have been detected in only five genera of haptophytes, namely *Emiliania* and *Gephyrocapsa* as calcifying species (coccolithophores), and *Isochrysis*, *Tisochrysis* and *Chrysotila* as non-calcifying species^11–15^. The alkenones are structurally robust^16,17^ and are often used in organic geochemical and palaeontological studies as biomarkers of the previous existence of coccolithophores.

One unique feature of alkenone molecules is *trans*-configuration carbon-carbon double bonds, whereas the fatty acids making up the lipid membranes typically have a *cis-*configuration^18,19^. The number of *trans*-type double bonds in alkenones increases or decreases, respectively, with decreases or increases in temperature during the growth of alkenone-producing haptophytes^14,17,20^. When *E*. *huxleyi* cells are grown at the optimum growth temperature (about 25 °C), they produce mainly di-unsaturated alkenones such as K_37:2_, K_38:2_, K_38:2_Et and K_39:2_. On the other hand, at low temperatures (about 10 °C), the cells produce more tri-unsaturated alkenones such as K_37:3_, K_38:3_, K_38:3_Et and K_39:3_^20^. Therefore, the ratio of di-unsaturated to tri-unsaturated alkenones extracted from sediment samples has been used to estimate the palaeotemperatures of the sea surface where alkenone-producing coccolithophores grew in past times and geological eras^21^. To reconstruct palaeotemperatures by using alkenones extracted from oceanic and lake sediments, many calibrations of the alkenone unsaturation index vs. growth temperature have been made experimentally by using various laboratory-cultured coccolithophores grown at different temperatures^10,22–24^.

Despite this active palaeoceanographic and palaeoclimatic research, the biosynthetic pathway of alkenones has not yet been fully studied. In our previous study, we showed that cerulenin, which inhibits the biosynthesis of C_16_–C_18_ fatty acids by inhibiting β-keto-acyl-ACP synthase (one of the moieties of fatty acid synthase), suppressed alkenone biosynthesis in *E*. *huxleyi* cells^25^. These results suggested that the alkenone biosynthesis pathway was located downstream of the fatty-acid elongation pathway^25^.

**Figure 1:**
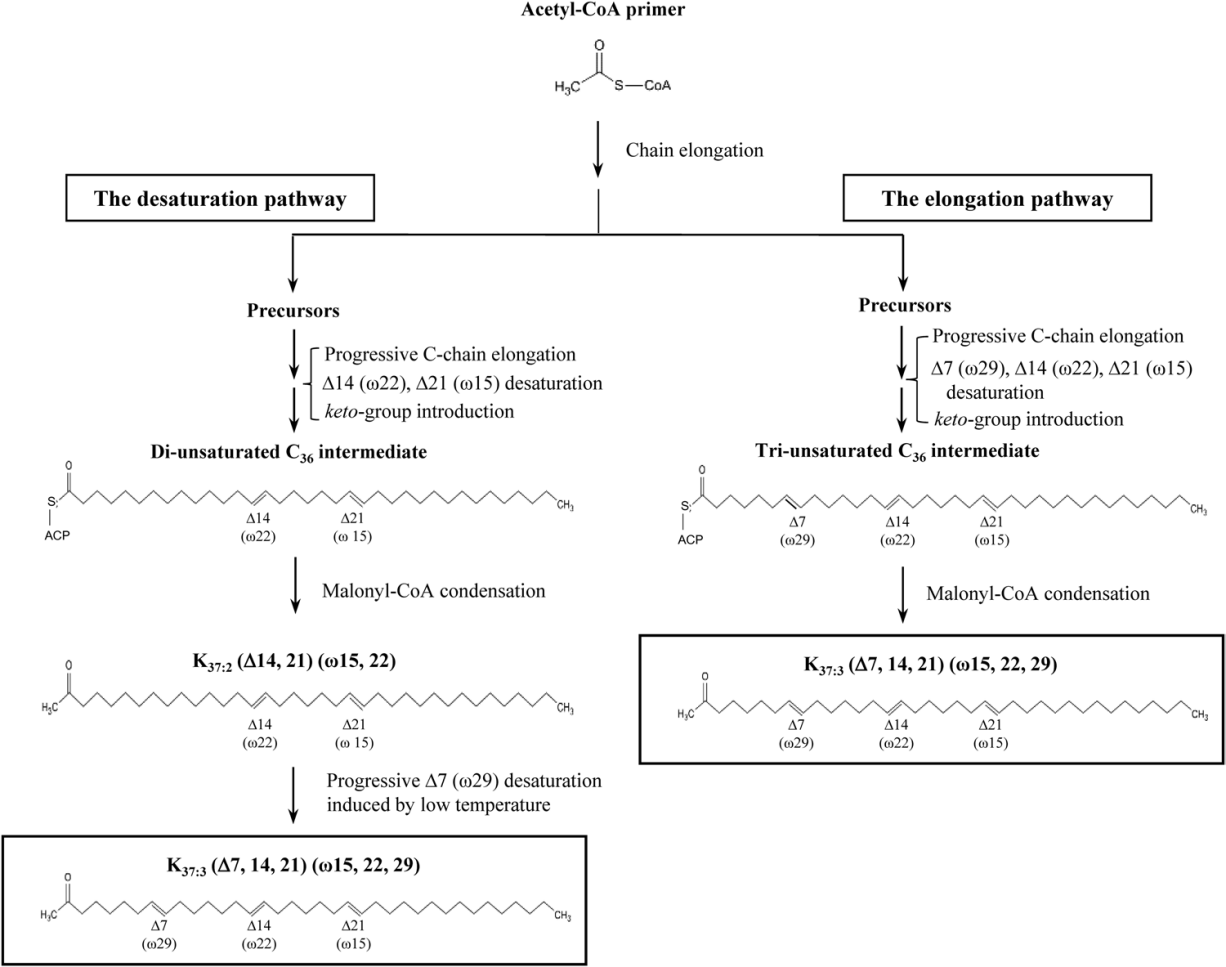
Schematic model of the predicted alternative pathways of K_37:3_ biosynthesis in the coccolithophore *Emiliania huxleyi*. The figure is based on the concept proposed in a previous report by Rontani *et al*.^26^.

From molecular and structural information on various alkenones isolated from living cells and from analyses of Black Sea sediments, Rontani *et al*.^26^ presented a schematic model for the whole biosynthetic pathway of alkenones. They hypothesised the following (Fig. 1): 1) the carbon chains of alkenone molecules are elongated in the fatty acid elongation pathway; 2) a methyl end is formed by the condensation of malonyl-CoA, and an ethyl end is formed by the condensation of methylmalonyl-CoA; 3) *trans*-unsaturation carbon-carbon double bonds are produced by putative desaturases in the final step of alkenone biosynthesis after the process of elongation, and these putative desaturases may be similar to the fatty-acid desaturase catalysing the canonical *cis*-unsaturation of fatty acids^26^.

Two hypotheses have been proposed regarding the production of tri-unsaturated alkenones (K_37:3_, K_38:3_, K_39:3_, and K_40:3_)^26^ (Fig. 1): 1) Di-unsaturated molecules (K_37:2_, K_38:2_, K_39:2_, and K_40:2_) are first synthesized via four reactions, namely chain elongation, malonyl-CoA condensation including decarboxylation and desaturation in sequence, and then the di-unsaturated molecules are processed to tri-unsaturated molecules by a desaturation reaction (**desaturation pathway**). 2) Various tri-unsaturated precursors are separately synthesized first, and then the chains of the precursors are separately elongated to produce individual molecules with longer chains (**elongation pathway**). In the latter case, two sub-pathways should exist for synthesizing either di- or tri-unsaturated alkenones separately, and small molecules of alkenone precursors (smaller than K_37:3_) should be found upstream of the pathways. In other words, in the second hypothesis, both di- and tri-unsaturated alkenones need to be independently synthesized via the metabolic pathways of chain elongation, desaturation, malonyl-CoA condensation and decarboxylation in parallel. However, as yet there is no experimental evidence for these metabolic intermediates or for enzymes that catalyse the reactions for alkenone biosynthesis.

Here, we performed a ^13^C-labelling experiment using *E*. *huxleyi* to determine which pathway actually functions in the synthesis of C_37_ tri-unsaturated alkenones (K_37:3_)—in other words, whether the desaturation-last hypothesis or the separate elongation hypothesis, or both, are correct. To reveal the metabolic path of K_37:3_ biosynthesis experimentally, *E*. *huxleyi* cells were first incubated with ^13^C-CO_2_ at 25 °C to produce only ^13^C-labelled C_37:2_ alkenones (^13^C-K_37:2_) without ^13^C-K_37:3_. The cells were then transferred rapidly to 15 °C to start the production of K_37:3_, and changes in the amounts of ^13^C-K_37:2_ and ^13^C-K_37:3_ were monitored.

## Results

**Figure 2:**
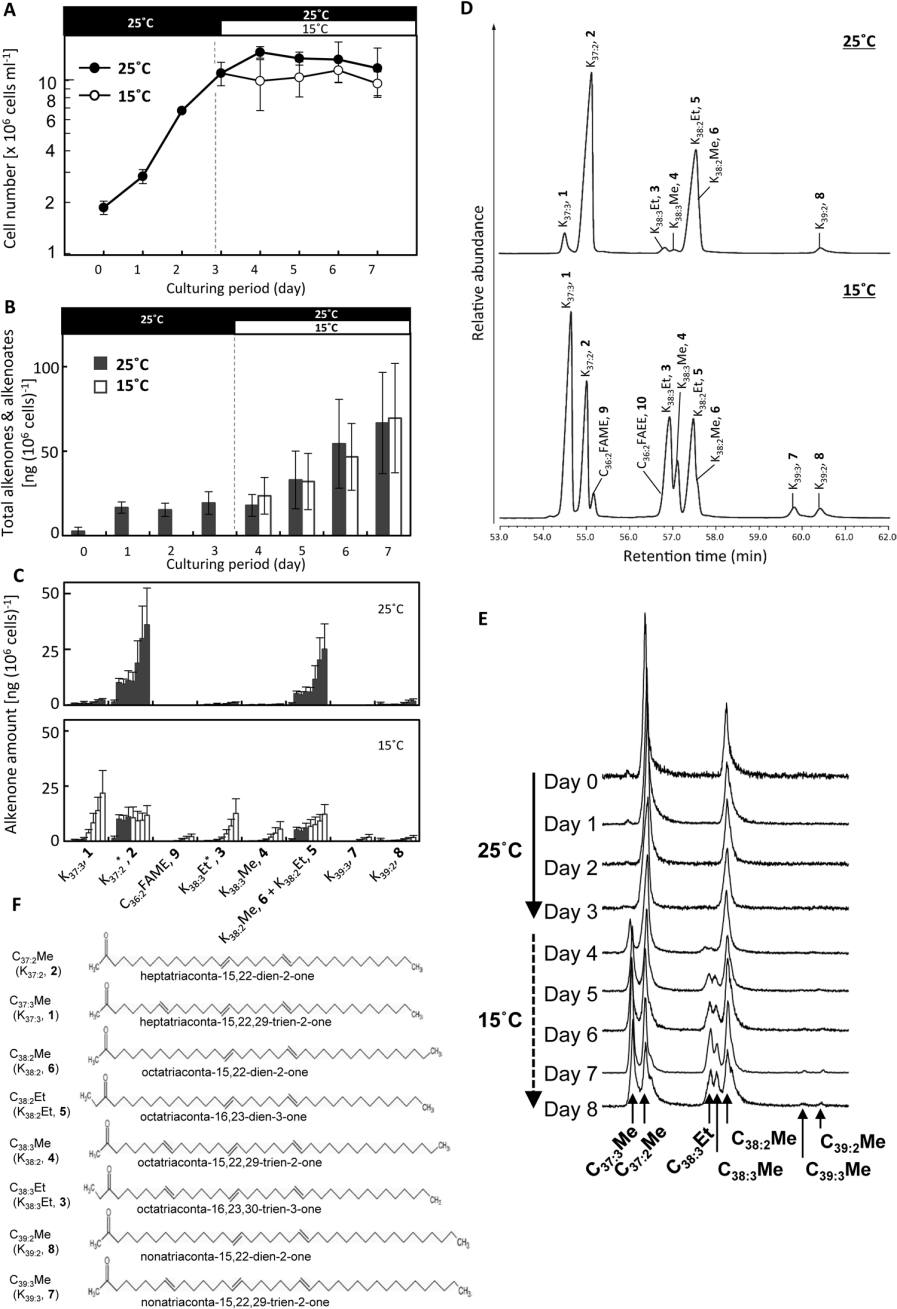
Changes in cell growth and alkenone composition during ^13^C-labelling experiments performed at different temperatures. (**A**) Cell growth profiles at 25 °C (black bar on top) and 15 °C (open bar on top). *Emiliania huxleyi* cells were first incubated with ^13^C-bicarbonate at 25 °C for 3 days. Then, half of the culture was transferred to 15 °C, and the other half was maintained at 25 °C. (**B**) Changes in the contents of whole alkenones and alkenoates at 25 °C (black bar on top) and 15 °C (open bar on top). (**C**) Changes in the contents of various components of alkenones and alkenoates (FAME) at 25 °C (black columns) and 15 °C (open columns) from days 0 to 7. *K_37:2_ and K_38:3_Et could have contained small amounts of C_36:2_FAME and C_36:2_FAEE, respectively, because of difficulties in separating these out by GC-FID. The bold numbers with compounds represent alkenone and alkenoate species shown in Fig. 2F. (**D**) GC-FID chromatogram patterns of alkenone and alkenoate compounds in cells grown at 25 °C (upper) and 15 °C (lower) and harvested on day 7. The number attached to each compound/peak is the same as in Fig. 2F. (**E**) Changes in the GC-FID chromatogram profiles of alkenones and alkenoates in cells grown at 25 °C and then transferred to 15 °C. (**F**) Chemical structural formulas of alkenones. Numbers in parenthesis are the same as in Fig. 2C,D. Each value represents the average of triplicate experiments. Error bars represent means ± SD.

For the ^13^C-labelling experiments, *E*. *huxleyi* cultures were first incubated with sodium ^13^C-bicarbonate (final concentration: 4 mM), which was added as a photosynthetic substrate, under continuous illumination by 20-W fluorescent lamps with an intensity of 100 μmoles/m^2^/s at 25 °C for 3 days (Fig. 2). Cells that had reached the late linear growth phase were then rapidly transferred to either 15 °C (for exposure to cold stress) or continued to be cultured at 25 °C (as controls). Cell growth stopped immediately at 15 °C, whereas the cells continued to grow a little further at 25 °C (Fig. 2A).

We detected eight alkenone derivatives (K_37:2_, K_37:3_, K_38:2_Me, K_38:2_Et, K_38:3_, K_38:3_Et, K_39:2_ and K_39:3_) and two alkenoate derivatives (C_36:2_-fatty acid methyl ester (FAME) and C_36:2_-fatty acid ethyl ester (FAEE)) in *E*. *huxleyi* CCMP2090 cells, although the C_39_ alkenones were very minor components (Fig. 2C–F, Table S1). The total amount of alkenones and alkenoates combined increased independently of temperature during the stationary growth phase, but it increased very little in the logarithmic growth phase (Fig. 2B).

The composition of alkenones alone changed markedly with temperature—especially the relative amounts between di- and tri-unsaturated molecules in C_37_–C_39_ alkenones (Fig. 2C–E, Fig. 2F for annotation of molecular species with molecular structures). At 25 °C, K_37:2_ was synthesized in abundance (97.2 ± 34.3 ng [10^6^ cells]^−1^ on days 0 to 3 and 249.0 ± 121.0 ng on days 3 to 7), whereas little K_37:3_ was produced (9.8 ± 5.5 ng [10^6^ cells]^−1^ on days 0 to 3, and 12.2 ± 0.2 ng on days 3 to 7). When the temperature was decreased rapidly to 15 °C, K_37:3_ was synthesised rapidly in large amounts (207 ± 86.9 ng [10^6^ cells]^−1^) over the four days from days 3 to 7), whereas little K_37:2_ was synthesised (11.4 ± 5.7 ng [10^6^ cells]^−1^) (Fig. 2, Table S1).

The response of C_38_ alkenones to cold stress showed a trend similar to that of C_37_ alkenones during 7 days (Fig. 2, Table S1). The cellular contents of K_37:3_ and K_38:3_Et increased ca. 20 (changed from 2.7 ± 1.7 to 54.9 ± 33.9) and ca. 26 times (changed from 4.9 ± 3.6 to 126.2 ± 66.1), respectively, at 15 °C during the four days from day 3 to day 7, whereas those of K_38:2_ and K_38:2_Et only doubled. The K_39:3_ content increased about 37 times (changed from 0.5 ± 0.7 to 18.3 ± 12.6) at 15 °C from days 3 to 7, whereas the K_39:2_ content increased only 5.6 times (changed from 3.0 ± 1.8 to 16.8 ± 9.7), although the C_39_ alkenone content was very low.

**Figure 3:**
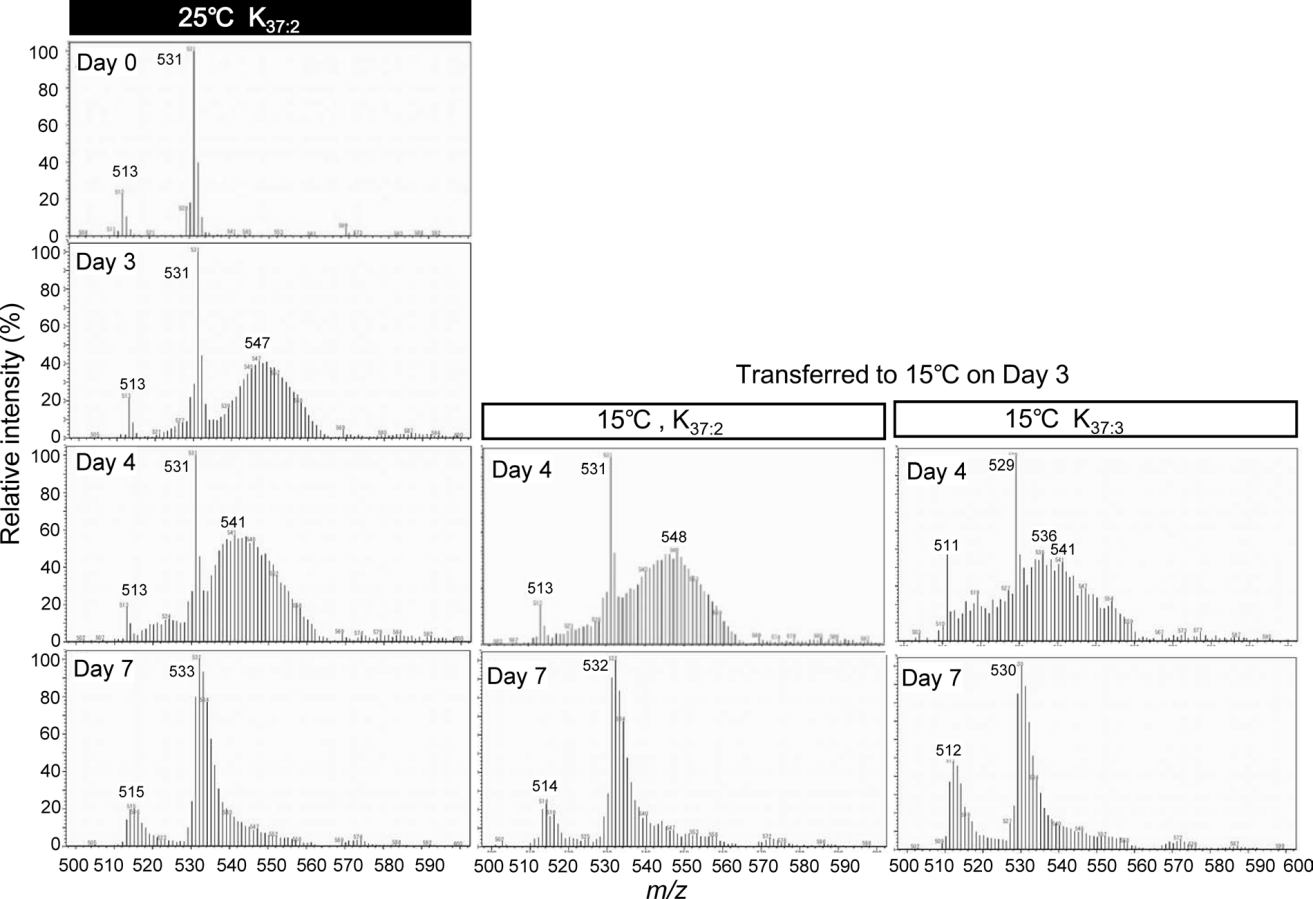
Mass spectrometric analysis over time during the ^13^C-labelling experiments. Sodium ^13^C-bicarbonate (final concentration, 4 mM) was added on day 0 of the labelling experiments. The ^13^C-labelling patterns of K_37:2_ alkenone in cells grown at 25 °C are shown for days 0, 3 and 4. The ^13^C-labelling patterns in cells grown at 15 °C on day 4 (24 h after the change to 15 °C temperature) are shown for K_37:2_ and the newly formed K_37:3_. See Fig. 2 for growth curves and changes in alkenone contents.

The mass spectrometric patterns of the ^13^C-labelling profiles of C_37_ alkenones were determined by gas chromatography – mass spectrometry (GC-MS) analysis (Fig. 3, Fig. S1 for detailed mass spectrometric profiles on time courses). On day 0, before the start of ^13^C-labelling, K_37:2_ could be quantified exactly at 531 (*m/z*) as a protonated form together with an *m/z* 513 signal derived from a K_37:2_-fragment upon GC-MS using isobutane for chemical ionization (Fig. S1, A). After the start of ^13^C-labelling at 25 °C, signals at >531 (*m/z*) increased markedly on days 1 to 3 (Fig. S1, A to C). After day 4, the signals at >531 (*m/z*) gradually decreased, with a shift to the *m/z* 513 signal in both K_37:2_ and K_37:3_, although the patterns differed depending on conditions and alkenone molecular species among K_37:2_ at 25 °C (Fig. S1, E–H), K_37:2_ at 15 °C (Fig. S1, I–L) and K_37:3_ at 15 °C (Fig. S1, M to P).

**Figure 4:**
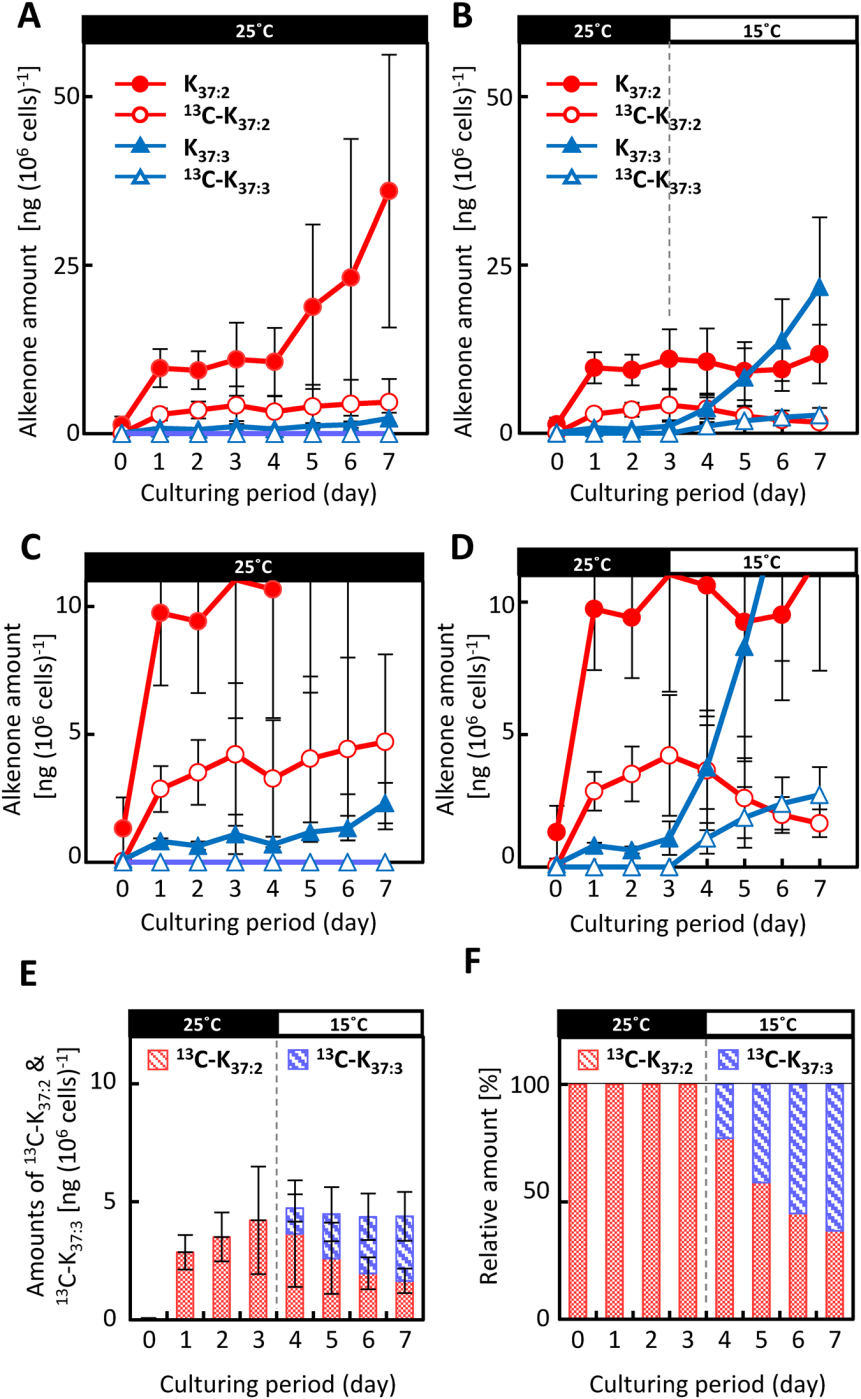
Changes in C_37_ alkenone composition and labelling patterns over time during the ^13^C-labelling experiments. C_37_ alkenones were quantified over time, and amounts are shown for total K_37:2_ (closed red circles), ^13^C-K_37:2_ (open red circles), total K_37:3_ (closed blue circles), and ^13^C-K_37:3_ (open blue circles) in cells grown at 25 °C (**A**) and transferred to 15 °C on day 3 (**B**). Magnifications of graphs (**A**) and (**B**) are shown as (**C**) and (**D**), respectively. Total C_37_ alkenones and compositions at 15 °C are shown as amounts (**E**) and relative amounts (**F**). Each value is the average of three independent experiments. Error bars represent means ± SD.

The *m/z* 568 signal corresponds to ^13^C-K_37:2_, in which all C molecules are labelled or substituted with ^13^C. Therefore, the ^13^C atom% values in K_37:2_ were calculated from the experimental data, with correction for the influence of the natural ^13^C/^12^C ratio in accordance with the method of Hamanaka *et al*.^27^. The ^13^C atom% values in K_37:2_ and K_37:3_ (namely, ^13^C-K_37:2_ (%) and ^13^C-K_37:3_ (%), respectively) were quantified by using the relative intensities of the *m/z* 531 and 529 signals, respectively (Table S2). The amounts of ^13^C-K_37:2_ and ^13^C-K_37:3_ were calculated from the GC-MS values of the ^13^C atom% and the values for the amount of each alkenone, as quantified by gas chromatography-flame ionisation detection (GCFID) (Table S2, Fig. 4).

The ^13^C-labelling efficiency of K_37:2_ in cells grown at 25 °C was 36.0% (^13^C atom%), meaning that 36.0% of the carbons in each K_37:2_ molecule were substituted with ^13^C. The maximum value was obtained on day 3 (Fig. 4, Table S2). When the temperature was maintained at 25 °C, the ^13^C-K_37:2_-value (^13^C atom%) gradually decreased after day 3 and reached 29.6% at 25 °C and 33.3% at 15 °C by day 4. Thereafter, the values continued to decrease and reached 13% at 25 °C and 14% at 15 °C by day 7 (Table S2).

The ^13^C-K_37:3_ content was negligible (below the detection limit) in the cells maintained at 25 °C for the whole period (Fig. 4A–D, Table S2). However, when the temperature was dropped to 15 °C on Day 3, the ^13^C-K_37:2_ content decreased linearly afterwards, whereas the ^13^C-K_37:3_ content increased; the stoichiometric balance indicated that the patterns of the two values mirrored each other (Fig. 4E, Table S2). Importantly, the sum of ^13^C-K_37:2_ plus ^13^C-K_37:3_ remained constant from Days 4 to 7 at the same level as that of ^13^C-K_37:2_ alone on day 3. In the experiment, ^13^C-pulse-labelling was performed by adding ^13^C-inorganic carbons as substrate first. And the labelling was continued until the cessation of increase in ^13^C-K_37:2_ showing the steady value where all inorganic carbons were depleted. Then, temperature was decreased to 15 °C for initiating the production of K_37:3._ This process provided conditions like a pulse-chase experiment. Under such conditions, there was no newly produced ^13^C-K_37:2_. As the experiment was performed as ^13^C-pulse-chase-like labelling experiment, the trend was more clearly seen in the pattern of change in the relative amounts of the ^13^C atom% values of ^13^C-K_37:2_ and ^13^C-K_37:3_ (Fig. 4F).

**Figure 5:**
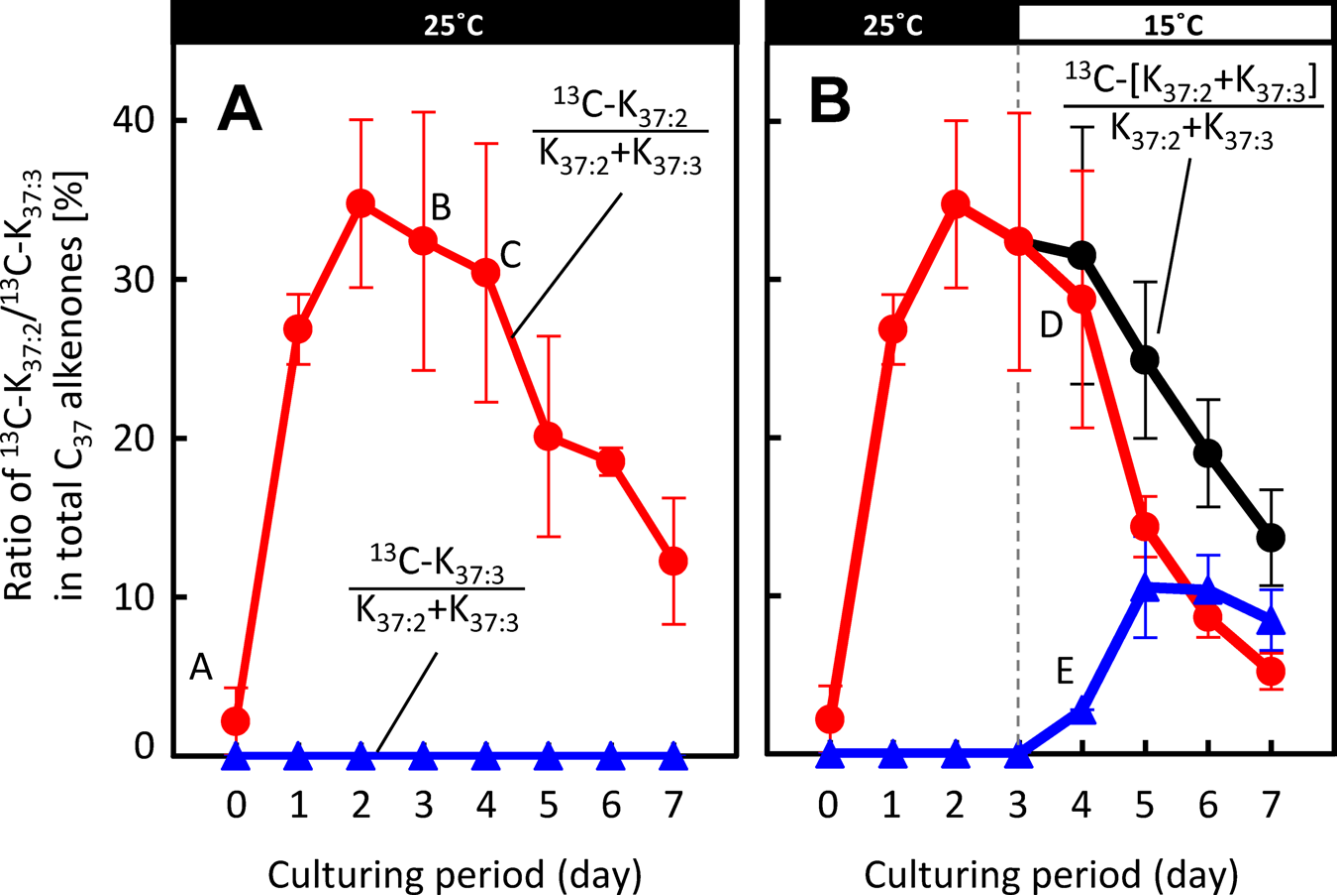
Changes in percentage of ^13^C-K_37:2_ and K_37:3_ to total C_37_ alkenones over time during the ^13^C-labelling experiments. The percentage of labelled K_37:2_ alkenones from *Emiliania huxleyi* cells grown at 25 °C (left) were compared with the percentages of labelled K_37:2,_ K_37:3_, and K_37:2_ + K_37:3_ alkenones from *E*. *huxleyi* cells transferred to 15 °C on day 3 (right). Red, blue and black lines are ^13^C-K_37:2_, ^13^C-K_37:3_ and ^13^C-[K_37:2_ + K_37:3_] as percentages of the total C_37_ alkenones, including all ^12^C- and ^13^C-labelled C_37_ alkenones. Cells used for analysis are the same as those in Fig. 2. For mass spectra, numbers of experiments, and explanations of error bars, see the captions to Figs 3 and 4.

We plotted changes in the ratios of ^13^C-K_37:2_ and ^13^C-K_37:3_ to total C_37_ alkenones (%) over time during the ^13^C-labelling experiments (Fig. 5). At 25 °C the ratio of ^13^C-K_37:2_ to total C_37_ alkenones clearly peaked on day 3 and then decreased gradually and linearly with time (Fig. 5A). At 15 °C the decrease in value was more pronounced. At this temperature the value of ^13^C-K_37:3_ clearly increased with the decrease in ^13^C-K_37:2_, in stoichiometric balance (Fig. 5B). The profile of the sum of ^13^C-K_37:2_ and ^13^C-K_37:3_ (^13^C-[K_37:2_ + K_37:3_], Fig. 5B) was very similar to that of ^13^C-K_37:2_ at 25 °C whereas the ratio of ^13^C-K_37:3_ to total C_37_ alkenones remained negligible (Fig. 5A)_._

## Discussion

In this study we intended to elucidate the mechanism of biosynthesis of tri-unsaturated alkenones such as K_37:3_ in the alkenone-producing haptophyte alga *E*. *huxleyi* CCMP2090 in the experiment by using a ^13^C-pulse-chase labelling technique. The experiments were designed to determine which pathway either the desaturation pathway or the elongation pathway dominated (see Fig. 1).

The principle of the ^13^C-labelling experiment was as follows: If K_37:3_ is directly synthesized from K_37:2_ via the desaturation pathway, the decrease in ^13^C-K_37:2_ should be compensated for a stoichiometric increase in ^13^C-K_37:3_ under cold-stress conditions, when the K_37:3_/K_37:2_ ratio is significantly increased. On the other hand, if ^13^C-K_37:3_ is synthesized via the elongation pathway, then the contents of ^13^C-K_37:2_ and ^13^C-K_37:3_ will change in a way different from that in the former pathway, without a match of the stoichiometry of the two molecules. Time course of ^13^C-labelling profiles of alkenones seem very similar. This pattern suggests that ^13^C-K_37:3_ is synthesized from ^13^C-K_37:2_, but not small precursors. In addition, the present results show that the degradation of K_37:2_and K_37:3_ is already initiated on day 7 (Fig. 3) although such degradation products and small alkenone molecules were not detected in this experiments.

Figure 4 clearly shows the typical mirror-image-like change in the contents of ^13^C-K_37:2_ and ^13^C-K_37:3_ when the sum of the contents of the two was maintained at the same level as the content of ^13^C-K_37:2_ before the start of the cold-triggered reaction. Figure 5 demonstrates that the production of ^13^C-labelled C_37_ alkenones ceased on day 3; thereafter, the previously produced ^13^C-alkenones were metabolised to other compounds. The data on ^13^C atom% strongly support the hypothesis that ^13^C-K_37:2_ was converted to ^13^C-K_37:3_ without the new production of ^13^C-K_37:2_ (Fig. 4D). This trend is also apparent in Fig. 5: the ^13^C atom% values of ^13^C-K_37:2_ at 25 °C and the sum of ^13^C-[K_37:2_ + K_37:3_] at 15 °C decreased in very similar ways.

In mass spectrometric analysis, ^13^C-signals between *m/z* 513 and *m/z* 540 include signals derived from fragments of ^13^C-K_37:2_ since ^13^C-K_37:2_ should give m/z > 531 since the complete molecule of ^12^C-K_37:2_ (Fig. 3, Fig. S1). The signal height of *m/z* 513 was 1/4 of *m/z* 531 signals and no detectable contaminants were detected in K_37:2_ by both separate TLC analysis (data not shown) and GC-FID analysis (see Fig. 2D). Therefore, we considered that the influence of signals derived from ^13^C-K_37:2_ fragments is unlikely to be large enough to create serious artifacts.

Finally, we concluded that K_37:3_ is synthesized from K_37:2_ by a desaturation reaction that is stimulated by cold stress via the desaturation pathway shown in Fig. 1. In accordance with the molecular structure of alkenones, the reaction should be catalysed by a putative Δ7 (ω29) alkenone desaturase, which should result in *trans*-type configuration.

Similar data on ^13^C-labelling profiles were also obtained for C_38_-alkenones, although the identification of various derivatives of K_38_-alkenones was not sufficiently accurate to perform a high-quality analysis: some of the peaks in the GC profiles overlapped (Fig. S2). Even under such conditions, the ^13^C-C_38_ alkenone profiles including K_38:2_Me + K_38:2_Et at 25 and 15 °C, K_38:2_Me at 15 °C and K_38:2_Et at 15 °C in the GC-MS analysis shown in Fig. S2 seemed similar to the ^13^C-C_37_ alkenone profiles shown in Fig. 3. We therefore considered that ^13^C-K_38:2_ molecules were produced at 25 °C, and then ^13^C-K_38:3_ molecules were synthesized by a desaturation reaction from the ^13^C-K_38:2_ molecules at 15 °C since the profile showed the decrease in ^13^C-K_38:2_ and the increase in ^13^C-K_38:3_ in balance (Fig. S2).

In microalgae, it is widely accepted that production of fatty-acid desaturases is induced by a downward shift in growth temperature and that these desaturases catalyse the desaturation of fatty acids in membrane lipids^28–30^. Most of the fatty-acid desaturases introduce a *cis*-configuration of carbon-carbon double bonds into the acyl chains conjugated with lipids or acyl carrier protein^29^.

The carbon-carbon double bonds found in alkenones are all of *trans*-configuration^18,19^. In regard to the formation of *trans*-type carbon-carbon double bonds in fatty acids, examples of the conversion reactions from *cis-* to *trans*-configuration by *cis*-*trans* isomerases are found in some heterotrophic bacteria^31^. Other types of fatty acid-desaturases that directly introduce a *trans*-type carbon-carbon double bond have been reported in the land plant *Arabidopsis thaliana*^32^ and insect *Epiphyas postvittana*^33^. Long-carbon-chain fatty acids are specifically found in sphingolipids—namely sphingosine, which has one or two carbon-carbon double bonds of *trans*-configuration that are directly introduced by the action of sphingolipid Δ4 and Δ8 desaturases in mice^34^ and in Arabidopsis^35^.

With this information as background, we tried to screen for orthologous genes in the *E*. *huxleyi* genomic sequence published by Read *et al*.^36^ by using a homology search of the amino-acid sequences of enzymes functioning in the formation of *trans*-type carbon-carbon double bonds. In the database, we succeeded in finding two genes encoding proteins homologous to a sphingolipid *trans*-Δ8 desaturase of Arabidopsis^35^ and to a *trans*-fatty acid desaturase of *Brassica rapa*^37^ in higher plants.

The structures of alkenones (i.e. their carbon length, functional moiety at the terminal end, and distance between the double bonds) differ from those of typical unsaturated fatty-acids. Therefore, the primary sequence and substrate specificity of alkenone desaturase, if it is identified in future, may differ from those of the canonical fatty-acid desaturases. Further functional and expression analyses of the genes will be necessary to conclude that the candidates are indeed alkenone-specific desaturases.

The acyl chain of an unsaturated fatty acid is bent at the *cis*-type carbon-carbon double bond position, and this bending alters the microenvironment around the lipids. Additionally, membrane fluidity is increased, because the melting point of the unsaturated fatty acids is decreased. In contrast, the *trans*-type carbon-carbon double bonds found in alkenones cause very little structural change, and the melting points of *trans*-type fatty acids are not much lower than those of *cis*-type fatty acids.

It is interesting to note that the number of *trans*-type carbon-carbon double bonds in alkenones increases with decreasing temperature, although alkenones are not membrane lipids^38^. In fact, alkenones have been shown experimentally to be stored in the lipid body as storage lipids in *E*. *huxleyi*^39^ and *Tisochrysis lutea*^40^, and the location is similar to that of triacylglycerols in other eukaryotic microalgae^41^. Alkenones stored in the lipid body are experimentally proved to function as energy storage, but there is temperature-dependent change in molecules. Namely, K_37:2_ and K_37:3_ are dominant molecules at high and low temperatures, respectively. Therefore, the cold-induced desaturation reaction should play very important role for carbon and energy storage especially at cold environment. However, it is still not perfectly clear why *trans*-type desaturation is induced with an almost linear responsive pattern in alkenones by cold stress.

The lipid body in the alkenone-producing haptophyte alga *T*. *lutea* was named the alkenone body because it consists primarily of alkenones, and it is surrounded by a single membrane derived from endoplasmic reticulum^40^. According to the evidence presented in this study, the newly synthesized ^13^C-alkenones are likely accumulated in the ^12^C-alkenone body; by Day 3 the ^13^C-atom% of K_37:2_ had reached 36% in cells grown at 25 °C (Fig. 5, Table S2). If di-unsaturated alkenones are converted to tri-unsaturated alkenones in the alkenone body, then the desaturation reaction likely occurs there. There is a strong need for further experimental evidence to prove the localization of the putative *trans*-type alkenone desaturase(s).

The results of the present study are supported by following two reports which proposed the presence of putative alkenone Δ7 (ω29) desaturases predicted by the analysis of haptophyte cells and natural samples. Rontani *et al*.^26^ predicted a schematic model of alkenone biosynthesis based on structural information on alkenones found previously in living cells and natural environments (Fig. 1). According to the hypothetical pathway, a predicted alkenone Δ7 (ω29) desaturase catalyses the introduction of a *trans*-type carbon-carbon double bond into the Δ7 (ω29) position of K_37:2_ (Δ14, 21) (ω15, 22) to synthesize K_37:3_ (Δ7, 14, 21) (ω15, 22, 29) (Fig. 1). Zhen *et al*.^42^ proposed that all the alkenone-producing haptophytes have the same Δ7, 14, 21, 28 desaturases and the formation of three methylene-interrupted double bonds results from β-oxidation of the alkenone intermediate prior to the final desaturation step in the shorter-chain alkenone (SCA) biosynthesis pathway. This proposal was based on the analysis of different alkenone isomers found in cultures and extreme environments, namely that Δ7-desaturation occurs from the di-unsaturated isomer prior to other modifications.

In this study, we proved the occurrence of the Δ7 (ω29) desaturase reaction experimentally, although the enzyme molecule of the predicted alkenone Δ7 (ω29) desaturase has not yet been identified. Namely, there is as yet no evidence of any other factors, such as enzymes, contributing to the reactions required to build the molecular structures of alkenones, such as carbon-chain elongation and *keto*-group formation. If we are to develop a comprehensive understanding of the whole pathway of alkenone biosynthesis, then we need further studies to identify the precursors or intermediates involved in alkenone biosynthesis. For this purpose, new analytical technology that combines genetic, biochemical, and physiological manipulation of metabolism needs to be developed for application to alkenone-producing haptophytes.

In the geological sciences, alkenones are frequently utilised as haptophyte biomarkers and palaeothermometers to estimate sea-surface temperatures on the basis of temperature-dependent changes in the degree of unsaturation of molecules. Therefore, the mechanism of desaturation of alkenones is very important in the construction of high-quality palaeothermometers and in elucidating the principles of alkenone palaeothermometers.

## Conclusion

Here, we used a ^13^C-tracer experiment to prove the mechanism by which K_37:3_ and K_38:3_ are synthesised from K_37:2_ and K_38:2_, respectively, by a Δ7 (ω29) desaturase reaction induced by cold stress in the coccolithophore *E*. *huxleyi*. Then, we concluded that ^13^C-K_37:3_ could be synthesized directly from ^13^C-K_37:2_ via the desaturation pathway by a cold-induced desaturation reaction. To our knowledge, this is the first experimental evidence of the existence of a desaturase reaction for alkenones in the alkenone-producing haptophyte *E*. *huxleyi*, although other hypothetical reactions via the elongation pathway cannot yet be fully discounted.

This study also provided very important information on the mechanism of the desaturation reaction of alkenones and how alkenone production is regulated by temperature change. The C_37_-alkenone unsaturation index (e.g., U^K′^_37_) is an important parameter that is used frequently in palaeotemperature estimation. Our findings are therefore the first step in elucidating the physiological and biochemical properties of alkenones and the basic temperature-responsive mechanism of the alkenone palaeothermometer.

## Experimental

### Organisms and culture conditions

Cells of *Emiliania huxleyi* (Lohman) Hay & Mohler CCMP 2090 were obtained from the National Center for Marine Algae and Microbiota (formerly the Culture Collection of Marine Phytoplankton), Bigelow Laboratory for Ocean Sciences, Maine, USA, in 2011 and stored in our laboratory. The strain was isolated in the South Pacific Ocean in 1991 and cloned as an axenic culture. Cells were grown in a flat-oblong glass vessel (either 500 mL or 2 L) containing artificial seawater (Marine Art SF-1, produced by Tomita Seiyaku Co. Ltd., Tokushima, Japan and distributed by Osaka Yakken Co. Ltd., Osaka, Japan) enriched with Erd-Schreiber’s seawater containing 10 nM sodium selenite instead of soil extracts (MA-ESM)^43^. The culture was continuously illuminated by white fluorescent lamps at an intensity of 100 µmol photons m^−2^ s^−1^ at 25 or 15 °C under aeration with sterilized air at a flow rate of 100 mL min^−1^. Growth of *E*. *huxleyi* was monitored as optical density at a wavelength of 750 nm (OD_750_) with a UV-1700 spectrophotometer (Shimadzu, Kyoto, Japan), by counting cell numbers under a microscope with a camera system (BX-50, Olympus, Tokyo, Japan), and with an automatic particle counter (CDX-1000×, Sysmex Corporation, Kobe, Japan).

### ^13^C-labelling experiment

For the ^13^C-labelling experiment, *E*. *huxleyi* cells were cultured in MA-ESM in which inorganic carbon was substituted with ^13^C. This medium was prepared by using the following procedure. First, HCl was added to the MA-ESM (pH 8.2) to reduce the pH of the medium to below 3. N_2_ gas was then bubbled through the medium for >1 h to eliminate dissolved inorganic carbons. Then, CO_2_-free NaOH solution (at an oversaturated concentration in water) was added under continuous N_2_ bubbling to adjust the pH to 8.2. Thereafter, sodium ^13^C-bicarbonate (CLM-441-PK, ^13^C 99%, 97% + chemical purity, Cambridge Isotope Laboratories, Inc., Tewksbury, MA, USA) was added (final concentration, 4 mM). The medium containing sodium ^13^C-bicarbonate was stored in a tightly capped glass bottle until use.

The *E*. *huxleyi* cells were pre-cultured in MA-ESM (500 mL) at 25 °C in the continuous light (100 μmol m^−2^ s^−1^) provided by 20w-fluorescent lamps. During pre-culture, OD_750_ was kept at < 0.4 to maintain the growth of the cells in logarithmic phase. After three pre-cultures, the cells were inoculated into MA-ESM (2 L) containing 4 mM sodium ^13^C-bicarbonate to obtain an OD_750_ of 0.1 and were then grown at 25 °C in the light for labelling metabolites with ^13^C (^13^C-pulse-labelling). The culture was continuously aerated with sterilized air containing ^12^CO_2_ at a flow rate of 100 mL min^−1^. The culture was maintained until when ^13^C-bicarbonate added was depleted. After 3 days, the culture was split into two. One part was maintained at 25 °C, and the other was grown at 15 °C for 4 more days under aeration with air containing ^12^CO_2_ for chasing the transfer of ^13^C into metabolites. This process provided conditions which is similar to a pulse-chase-experiment. Fifty millilitres of cell culture was transferred every 24 h into a sterilized plastic tube and a detergent Tween 20 was added at 0.01% of the final concentration for getting clear cell pellets in the tube without any damage of cells after weak centrifugation. The cultures were then centrifuged (800 *g* for 10 min, 4 °C) and 40 mL of the supernatant was removed. After being resuspended, the cells were centrifuged (800 *g* for 5 min, 4 °C) and then supernatants were removed completely. Cell pellets were resuspended in methanol (5 mL) and disrupted by ultrasonication (UD-201, Tomy Seiko, Tokyo) for 5 min at the device’s duty 70 setting on ice. The suspensions were stored at −80 °C until use for lipid extraction as described below and also according to previous reports^44,45^.

### Extraction of lipids and alkenones

Stored sample suspensions were centrifuged (3000 *g* for 5 min, 4 °C), and the resultant supernatants were transferred to new glass tubes. The precipitate was then extracted with methanol-dichloromethane (1:1, v/v; 5 mL) followed by dichloromethane (5 mL). At each extraction, the supernatant fraction was transferred and combined with the others in the same glass tube after centrifugation (3000 *g* for 5 min, 4 °C). After the addition of 0.1% *n*-triacontane (C_30_H_62_) dissolved in 100 μL of *n*-hexane was added as an internal standard for GC analysis. Thereafter, deionized water (25 mL) and saturated sodium chloride solution (5 mL) was added, the tubes were capped, and the contents mixed by voltexing for 5 min at room temperature.

After the solution had been left for 5 min to separate into water (upper) and dichloromethane (lower) layers, the dichloromethane layer was carefully collected with a Pasteur pipette. This layer was applied to a sodium-sulphate-packed column (1 g), and the eluate was collected into a new glass tube. After the addition of dichloromethane (5 mL) to the upper, aqueous layer, the contents were vigorously mixed for 5 min and allowed to separate. The dichloromethane (lower) layer was applied to the same sodium-sulphate-packed column for full extraction of lipids. The solvents were soft-evaporated under reduced pressure in a rotary evaporator (BÜCHI Labortechnik, Flawil, Switzerland). After the addition of *n*-hexane (2 mL), the solvents were completely evaporated. After being dissolved in *n*-hexane (2 ml), lipids were applied to a silica gel column (1 g silica) and eluted first with *n*-hexane (4 ml), then with *n*-hexane-ethyl acetate (95:5, v/v; 4 ml), and finally with *n*-hexane-ethyl acetate (90:10, v/v; 4 ml). The combined fraction of the four eluates, containing lipids such as ketones, esters, alkenes and alkanes, was completely evaporated, and then the residue was dissolved in *n*-hexane (1.5 mL). Each lipid solution was stored in teflon-lined glass vials at −20 °C until use.

### GC analysis

Various components of lipids were quantified by using a GC-FID system. The system consisted of a GC-2014 (Shimadzu) equipped with a CP-Sil5 CB capillary column (length, 50 m; internal diameter, 0.32 mm; Agilent Technologies, Santa Clara, CA). He gas was used as a carrier at a constant flow rate of 1.25 mL min^−1^ in split-less mode. The temperature was programmed as follows: 60 °C for 1.5 min, increased linearly to 130 °C at a rate of 20 °C min^−1^, further increased linearly to 300 °C at a rate of 4 °C min^−1^, then held constant at 300 °C for 25 min. The amount of each alkenone and alkenoate species in Table S1 was calculated based on the species’ peak area on the chromatograms compared with that of the internal standard, *n*-triacontane, and then normalized on the basis of cell number (for GC-FID profiles, see Fig. 2).

### GC-MS analysis

For GC-MS analysis, as described by Hamanaka *et al*.^27^, chemical ionization (CI) with isobutane as the reagent gas was used to obtain the quasi-molecular ion peaks for each C_37_ alkenone species (1 and 2), allowing us to evaluate the frequency of stable isotopic carbon in the alkenones.

To calculate the percentage ^13^C content (^13^C atom%) in each alkenone species, a GC-MS system composed of a GCMS-QP2010 device (Shimadzu) attached to a CP-Sil5 CB column (Agilent Technologies) was used. The flow rate of He gas as a carrier was 1.25 mL min^−1^. Compounds were analysed by the CI method using isobutane gas (with a pressure of 130 kPa) under the following conditions: ionization voltage, 60 eV; emission current, 50 μA; ionization temperature, 230 °C. Mass spectra at *m*/*z* 500 to 600 were scanned every 0.1 s. The column temperature was elevated from 150 to 320 °C at a rate of 5 °C min^−1^ and then kept at 320 °C for 20 min.

The ^13^C atom% of each alkenone was calculated from the ratios of isotopic ion peaks relative to the quasi molecular ion peak, as per the method of Kouchi^46^. Discrimination of ^13^C was not considered. The calculation is described here briefly. The quasi-molecular peak for ionized-K_37:2_ (2) [K_37:2_ + H^+^] was observed at *m*/*z* 531 (Fig. 2A). A K_37:2_ (2) molecule containing n^13^C [K_37:2_ + H^+^  + n] (n = number of ^13^C) was detected at *m*/*z* 531 + n. If all carbons in a molecule of K_37:2_ (2) were substituted with ^13^C, the *m*/*z* value was detectable at 568. The relative ratios of each quasi-molecular ion peak at *m*/*z* values in the ranges of 531 to 568 for K_37:2_ (2) and 529 to 566 for K_37:3_ (1) (Fig. 2) were compared with the relative ratios of the isotopic ion peaks for each alkenone species, which can be calculated theoretically according to previous report^24^.

The ^13^C atom% in K_37:2_ (2) and K_37:3_ (1) (determined by GC-MS analysis) and the absolute amount of each alkenone species (1 and 2) (determined by GC-FID) were used to calculate the amounts of ^13^C-labelled alkenones. Note that the mass spectra of K_37:2_ (2) before ^13^C-labelling represented the natural carbon isotope ratio (^12^C/^13^C), namely about 4.2% of the ^13^C atom%. (See day 0 in Table S2.)

## Electronic supplementary material

Supplementary Infomation

## References

[1] McIntyre, A. & Bé, A. W. H. Modern coccolithophoridae of the Atlantic ocean—I. Placoliths and cyrtoliths. *Deep Sea Res*. *Oceanograph. Abstracts* **14**, 561–597 (1967).

[2] Okada H, Honjo S (1973). The distribution of oceanic coccolithophorids in the Pacific. *Deep Sea Res*. Oceanograph. Abstracts.

[3] Orr JC (2005). Anthropogenic ocean acidification over the twenty-first century and its impact on calcifying organisms. Nature.

[4] Winter, A., Jordan, R. & Roth, P. Biogeography of living coccolithophores in ocean waters. In: Winter A. & Siesser W. G. (Eds.), Coccolithophores. Cambridge University Press, Cambridge, UK, pp. 161–177 (1994).

[5] Brown CW, Yoder JA (1994). Distribution pattern of coccolithophorid blooms in the western North Atlantic Ocean. Cont. Shelf Res..

[6] Feely RA (2004). Impact of anthropogenic CO_2_ on the CaCO_3_ system in the oceans. Science.

[7] Harada N (2012). Enhancement of coccolithophorid blooms in the Bering Sea by recent environmental changes. Global Biogeochem. Cycles.

[8] Fukuda S, Suzuki Y, Shiraiwa Y (2014). Difference in physiological responses of growth, photosynthesis and calcification of the coccolithophore *Emiliania huxleyi* to acidification by acid and CO_2_ enrichment. Photosynth. Res..

[9] Volkman JK, Eglinton G, Corner EDS, Forsberg TEV (1980). Long-chain alkenes and alkenones in the marine coccolithophorid *Emiliania huxleyi*. Phytochemistry.

[10] Prahl FG, Muehlhausen LA, Zahnle DL (1988). Further evaluation of long-chain alkenones as indicators of paleoceanographic conditions. Geochim. Cosmochim. Acta.

[11] Marlowe IT (1984). Long chain (n-C_37_–C_39_) alkenones in the Prymnesiophyceae. Distribution of alkenones and other lipids and their taxonomic significance. Brit. Phycol. J..

[12] Rontani J-F, Prahl FG, Volkman JK (2006). Characterization of unusual alkenones and alkyl alkenoates by electron ionization gas chromatography/mass spectrometry. Rapid Commun. Mass Spectrom..

[13] Conte MH, Thompson A, Eglinton G, Green JC (1995). Lipid biomarker diversity in the coccolithophorid *Emiliania huxleyi* (Prymnesiophyceae) and the related species *Gephyrocapsa oceanica*. J. Phycol..

[14] Volkman JK, Barrett SM, Blackburn SI, Sikes EL (1995). Alkenones in *Gephyrocapsa oceanica*: Implications for studies of paleoclimate. Geochim. Cosmochim. Acta.

[15] Rontani J-F, Beker B, Volkman JK (2004). Long-chain alkenones and related compounds in the benthic haptophyte *Chrysotila lamellosa* Anand HAP 17. Phytochemistry.

[16] Prahl FG, de Lange GJ, Lyle M, Sparrow MA (1989). Post-depositional stability of long-chain alkenones under contrasting redox conditions. Nature.

[17] Pan H, Sun M-Y (2011). Variations of alkenone based paleotemperature index (U^K′^_37_) during *Emiliania huxleyi* cell growth, respiration (auto-metabolism) and microbial degradation. Org. Geochem..

[18] Rechka JA, Maxwell JR (1988). Unusual long chain ketones of algal origin. Tetrahedron Lett..

[19] Volkman JK, Burton HR, Everitt DA, Allen DI (1988). Pigment and lipid compositions of algal and bacterial communities in Ace Lake, Vestfold Hills, Antarctica. Hydrobiologia.

[20] Prahl FG, Wakeham SG (1987). Calibration of unsaturation patterns in long-chain ketone compositions for palaeotemperature assessment. Nature.

[21] Brassell SC, Eglinton G, Marlow IT, Pflaumann U, Sarnthein M (1986). Molecular stratigraphy: a new tool for climatic assessment. Nature.

[22] Ono M, Sawada K, Shiraiwa Y, Kubota M (2012). Changes in alkenone and alkenoate distributions during acclimatization to salinity change in *Isochrysis galbana:* Implication for alkenone-based paleosalinity and paleothermometry. Geochem. J..

[23] Sun Q, Chu G, Liu G, Li S, Wang X (2007). Calibration of alkenone unsaturation index with growth temperature for a lacustrine species, *Chrysotila lamellosa* (Haptophyceae). Org. Geochem..

[24] Nakamura H, Sawada K, Araie H, Suzuki I, Shiraiwa Y (2014). Long chain alkenes, alkenones and alkenoates produced by the haptophyte alga *Chrysotila lamellosa* CCMP1307 isolated from a salt marsh. Org. Geochem..

[25] Shiraiwa, Y., Kubota, M., Sorrosa, J. M. & Wettstein-Knowles, P. von. Alkenone synthesis in *Emiliania huxleyi* probed with radiolabeled substrate and a fatty acid synthesis inhibitor. In: Saxena, N. (Eds.), Recent Advances in Marine Science and Technology, 2004, PACON International, Hawaii, pp. 27–36 (2005).

[26] Rontani J-F, Prahl FG, Volkman JK (2006). Re-examination of the double bond positions in alkenones and derivatives: Biosynthetic implications. J. Phycol..

[27] Hamanaka J, Sawada K, Tanoue E (2000). Production rates of C_37_ alkenones determined by ^13^C-labeling technique in the euphotic zone of Sagami Bay, Japan. Org. Geochem..

[28] Russell NJ (1984). Mechanisms of thermal adaptation in bacteria: blueprints for survival. Trends Biochem. Sci..

[29] Los DA, Murata N (1998). Structure and expression of fatty acid desaturases. Biochim. Biophys. Acta - Lipids & Lipid Metabolism.

[30] Guschina IA, Harwood JL (2006). Lipids and lipid metabolism in eukaryotic algae. Prog. Lipid Res..

[31] Heipieper HJ, Meinhardt F, Segura A (2003). The *cis*–*trans* isomerase of unsaturated fatty acids in *Pseudomonas* and *Vibrio*: biochemistry, molecular biology and physiological function of a unique stress adaptive mechanism. FEMS Microbiol. Lett..

[32] Gao J (2009). Fatty Acid Desaturase 4 of *Arabidopsis* encodes a protein distinct from characterized fatty acid desaturases. Plant J..

[33] Liu W, Jiao H, Murray NC, O’Connor M, Roelofs WL (2002). Gene characterized for membrane desaturase that produces (*E*)-11 isomers of mono- and diunsaturated fatty acids. Proc. Natl. Acad. Sci. USA.

[34] Beauchamp E (2007). Myristic acid increases the activity of dihydroceramide Δ4-desaturase 1 through its N-terminal myristoylation. Biochimie.

[35] Chen M, Markham JE, Cahoon EB (2012). Sphingolipid Δ8 unsaturation is important for glucosylceramide biosynthesis and low-temperature performance in *Arabidopsis*. Plant J..

[36] Read BA (2013). Pan genome of the phytoplankton *Emiliania* underpins its global distribution. Nature.

[37] Li S-F (2012). Isolation and functional characterization of the genes encoding Δ8-sphingolipid desaturase from *Brassica rapa*. J. Genet. Genomics.

[38] Eltgroth ML, Watwood RL, Wolfe GV (2005). Production and cellular localization of neutral long-chain lipids in the haptophyte algae *Isochrysis galbana* and *Emiliania huxleyi*. J. Phycol..

[39] Tsuji Y, Yamazaki M, Suzuki I, Shiraiwa Y (2015). Quantitative analysis of carbon flow into photosynthetic products functioning as carbon storage in the marine coccolithophore, *Emiliania huxleyi*. Mar. Biotechnol..

[40] Shi Q (2015). Proteomic analysis of lipid body from the alkenone-producing marine haptophyte alga *Tisochrysis lutea*. Proteomics.

[41] Murphy DJ (2001). The biogenesis and functions of lipid bodies in animals, plants and microorganisms. Prog. Lipid Res..

[42] Zheng Y, Dillon JT, Zhang Y, Huang Y (2016). Discovery of alkenones with variable methylene-interrupted double bonds: implications for the biosynthetic pathway. J. Phycol..

[43] Danbara A, Shiraiwa Y (1999). The requirement of selenium for the growth of marine coccolithophorids, *Emiliania huxleyi*, *Gephyrocapsa oceanica* and *Helladosphaera* sp. (Prymnesiophyceae). Plant Cell Physiol..

[44] Sawada K, Handa N, Shiraiwa Y, Danbara A, Montani S (1996). Long-chain alkenones and alkyl alkenoates in the coastal and pelagic sediments of the northwest North Pacific, with special reference to the reconstruction of *Emiliania huxleyi* and *Gephyrocapsa oceanica* ratios. Org. Geochem..

[45] Sawada K, Shiraiwa Y (2004). Alkenone and alkenoic acid compositions of the membrane fractions of *Emiliania huxleyi*. Phytochemistry.

[46] Kouchi H (1982). Direct analysis of ^13^C abundance in plant carbohydrates by gas chromatography-mass spectrometry. J. Chromatogr. A.

