# Supplementary material for: Cold-induced metabolic conversion of haptophyte di- to tri-unsaturated C37 alkenones used as palaeothermometer molecules
Source: Sci Rep. 2018 Feb 2;8:2196. doi: 10.1038/s41598-018-20741-2 (PMC5797101; doi:10.1038/s41598-018-20741-2)

1. Supplementary materials:

**Supplementary information for:**

# Cold-induced metabolic conversion of haptophyte di- to tri-unsaturated $C_{37}$ alkenones used as palaeothermometer molecules

Eri Kitamura[1,†], Tomonori Kotajima[1,2], Ken Sawada[3,4], Iwane Suzuki[2,5] & Yoshihiro Shiraiwa[2,5*]

[1]Graduate School of Life and Environmental Sciences, University of Tsukuba, 1-1-1 Tennodai, Tsukuba, Ibaraki, 305-8572 Japan

[2]Faculty of Life and Environmental Sciences, University of Tsukuba, 1-1-1 Tennodai, Tsukuba, Ibaraki, 305-8572 Japan

[3]Department of Natural History Sciences, Faculty of Science, Hokkaido University, N10W8, Kita-ku, Sapporo 060-0810, Japan

[4]CREST, JST, N10W8, Kita-ku, Sapporo 060-0810, Japan

[5]CREST, JST, 1-1-1 Tennodai, Tsukuba, Ibaraki, 305-8572 Japan

*Corresponding author: emilhux@biol.tsukuba.ac.jp, tel +81-29-853-4668, fax +81-29-853-6614

**Table S1. Tabulated data of alkenone composition over time during the $^{13}C$-labeling experiments.**

*E. huxleyi* cultures were inoculated with labeled sodium [$^{13}C$]bicarbonate at time zero at 25 ˚C. After three days, half of the culture was transferred to 15 ˚C. The results of these labeling experiments are shown over time for alkenone and alkenoate (FAME) species. Each value (Mean ± SD) represents the average of triplicate experiments. *Values of $K_{37:2}$ and $K_{38:3}Et$ contains a slight amount of $C_{36:2}FAME$ and $C_{36:2}FAEE$, respectively. -, not tested.

| Alkenone species | Growth tempera-ture | Alkenone amount [ng ($10^6$ cells)$^{-1}$] | | | | | | | |
|---|---|---|---|---|---|---|---|---|---|
| | | Day 0 | Day 1 | Day 2 | Day 3 | Day 4 | Day 5 | Day 6 | Day 7 |
| $K_{37:3}$ | 25˚C | 1.1 ± 0.8 | 8.2 ± 1.0 | 6.4 ± 1.3 | 10.9 ± 6.3 | 6.9 ± 0.9 | 11.8 ± 3.1 | 18.4 ± 6.1 | 23.1 ± 6.5 |
| | 15˚C | - | - | - | - | 37.7 ± 15.8 | 83.2 ± 43.3 | 138.8 ± 60.6 | 217.9 ± 102.7 |
| $K_{37:2}$ * | 25˚C | 13.2 ± 9.9 | 100.2 ± 20.0 | 94.2 ± 22.9 | 110.4 ± 44.2 | 106.7 ± 41.0 | 188.3 ± 99.6 | 298.1 ± 145.5 | 359.4 ± 165.2 |
| | 15˚C | - | - | - | - | 106.3 ± 49.4 | 92.5 ± 43.2 | 95.2 ± 32.2 | 117.7 ± 43.7 |
| $C_{36:2}FAME$ | 25˚C | 0.0 ± 0.0 | 0.0 ± 0.0 | 0.2 ± 0.2 | 0.5 ± 0.4 | 0.3 ± 0.3 | 0.1 ± 0.1 | 0.5 ± 0.6 | 0.1 ± 0.2 |
| | 15˚C | - | - | - | - | 1.7 ± 1.4 | 7.7 ± 5.4 | 13.9 ± 8.4 | 21.4 ± 11.3 |
| $K_{38:3}Et$ * | 25˚C | 0.2 ± 0.2 | 2.5 ± 1.0 | 2.1 ± 1.0 | 4.9 ± 3.6 | 2.6 ± 1.1 | 4.9 ± 2.6 | 8.0 ± 3.8 | 10.1 ± 4.6 |
| | 15˚C | - | - | - | - | 12.2 ± 6.6 | 33.4 ± 19.0 | 69.2 ± 30.6 | 126.2 ± 66.1 |
| $K_{38:3}Me$ | 25˚C | 0.1 ± 0.2 | 1.7 ± 0.5 | 1.4 ± 0.3 | 2.7 ± 1.7 | 1.3 ± 0.2 | 2.2 ± 0.7 | 3.1 ± 1.2 | 4.4 ± 1.5 |
| | 15˚C | - | - | - | - | 6.1 ± 4.2 | 18.3 ± 13.8 | 36.7 ± 24.8 | 54.9 ± 33.9 |
| $K_{38:2}Et$ + $K_{38:2}Me$ | 25˚C | 6.0 ± 5.2 | 51.4 ± 12.0 | 45.9 ± 14.1 | 60.2 ± 21.5 | 58.6 ± 20.7 | 115.6 ± 61.2 | 201.6 ± 100.3 | 250.8 ± 113.3 |
| | 15˚C | - | - | - | - | 67.8 ± 30.0 | 75.0 ± 34.4 | 91.6 ± 29.7 | 122.5 ± 43.7 |
| $K_{39:3}$ | 25˚C | 0.0 ± 0.0 | 0.0 ± 0.0 | 0.0 ± 0.0 | 0.5 ± 0.7 | 0.1 ± 0.1 | 0.1 ± 0.1 | 0.5 ± 0.6 | 1.1 ± 0.6 |
| | 15˚C | - | - | - | - | 1.0 ± 0.6 | 3.9 ± 3.9 | 10.0 ± 6.2 | 18.3 ± 12.6 |
| $K_{39:2}$ | 25˚C | 5.6 ± 7.9 | 2.1 ± 0.2 | 2.0 ± 1.3 | 3.0 ± 1.8 | 2.2 ± 1.6 | 6.8 ± 5.2 | 13.4 ± 9.2 | 17.4 ± 11.1 |
| | 15˚C | - | - | - | - | 3.2 ± 2.4 | 6.3 ± 5.2 | 10.8 ± 5.6 | 16.8 ± 9.7 |
| **Total** | 25˚C | 26.1 ± 23.2 | 166.1 ± 33.4 | 152.3 ± 39.6 | 193.1 ± 66.9 | 178.6 ± 64.7 | 329.7 ± 170.7 | 543.5 ± 262.8 | 666.4 ± 299.8 |
| | 15˚C | - | - | - | - | 235.9 ± 108.6 | 320.4 ± 166.6 | 466.3 ± 197.7 | 695.6 ± 323.6 |

**Table S2 Percent of $^{13}C$–labelled $C_{37}$ alkenones during the $^{13}C$-labelling experiments of *E. huxleyi* CCMP2090 cells at 25˚C and 15˚C.**

Changes in $^{13}C$ atom% of $C_{37}$ alkenones were calculated when the cells grown at 25 ˚C for 3 days were transferred from 25 to 15 and 25 ˚C (control) on Day 3. Each value (Mean ± SD) represents the average of triplicate experiments. N.D., not detected. -, not tested.

| Growth temperature | Alkenone species | | Day 0 | Day 1 | Day 2 | Day 3 | Day 4 | Day 5 | Day 6 | Day 7 |
|---|---|---|---|---|---|---|---|---|---|---|
| 25˚C | $K_{37:2}$ | $^{13}C$ atom% (%) | 4.2 ± 1.6 | 29.2 ± 2.3 | 37.2 ± 5.1 | 36.0 ± 8.2 | 29.6 ± 9.6 | 21.6 ± 6.7 | 16.6 ± 5.3 | 13.1 ± 4.2 |
| | | $^{13}C$-$K_{37:2}$ [ng $(10^6$ cells$)^{-1}$] | 0.4 ± 0.3 | 28.6 ± 7.3 | 35.1 ± 10.4 | 42.1 ± 22.8 | 32.7 ± 18.6 | 40.5 ± 26.2 | 44.2 ± 29.2 | 47.0 ± 27.9 |
| | $K_{37:3}$ | $^{13}C$ atom% (%) | N.D. | N.D. | N.D. | N.D. | N.D. | N.D. | N.D. | N.D. |
| | | $^{13}C$-$K_{37:3}$ [ng $(10^6$ cells$)^{-1}$] | N.D. | N.D. | N.D. | N.D. | N.D. | N.D. | N.D. | N.D. |
| 15˚C | $K_{37:2}$ | $^{13}C$ atom% (%) | - | - | - | - | 33.3 ± 8.7 | 27.5 ± 5.5 | 20.9 ± 3.3 | 14.4 ± 2.2 |
| | | $^{13}C$-$K_{37:2}$ [ng $(10^6$ cells$)^{-1}$] | - | - | - | - | 36.5 ± 22.6 | 26.0 ± 15.1 | 19.7 ± 6.8 | 16.5 ± 5.2 |
| | $K_{37:3}$ | $^{13}C$ atom% (%) | - | - | - | - | 27.9 ± 5.9 | 22.0 ± 4.9 | 17.7 ± 3.5 | 13.3 ± 3.5 |
| | | $^{13}C$-$K_{37:3}$ [ng $(10^6$ cells$)^{-1}$] | - | - | - | - | 10.8 ± 5.8 | 18.7 ± 11.5 | 24.0 ± 9.9 | 27.3 ± 10.4 |

**Figure S1 Mass spectrometric analysis over time during the $^{13}$C-labelling experiments.**

Sodium $^{13}$C-bicarbonate (final concentration, 4 mM) was added on day 0 of the labelling experiments. The $^{13}$C-labelling patterns of $K_{37:2}$ alkenone in cells grown at 25 ºC are shown for days 0 (A), 1 (B), 2 (C) and 3 (D) (exactly 24 h intervals). Thereafter, a half of the cells were transferred to 15 ºC on day 3. The $^{13}$C-labelling patterns in cells grown at 25 ºC and 15 ºC on day 4, 5, 6 and 7 (24 h intervals) are shown for $K_{37:2}$ (E-F at 25 ºC and I-L at 15 ºC, respectively) and the newly formed $K_{37:3}$ (M-P at 15 ºC)). See Fig. 2 for growth curves and changes in alkenone contents.

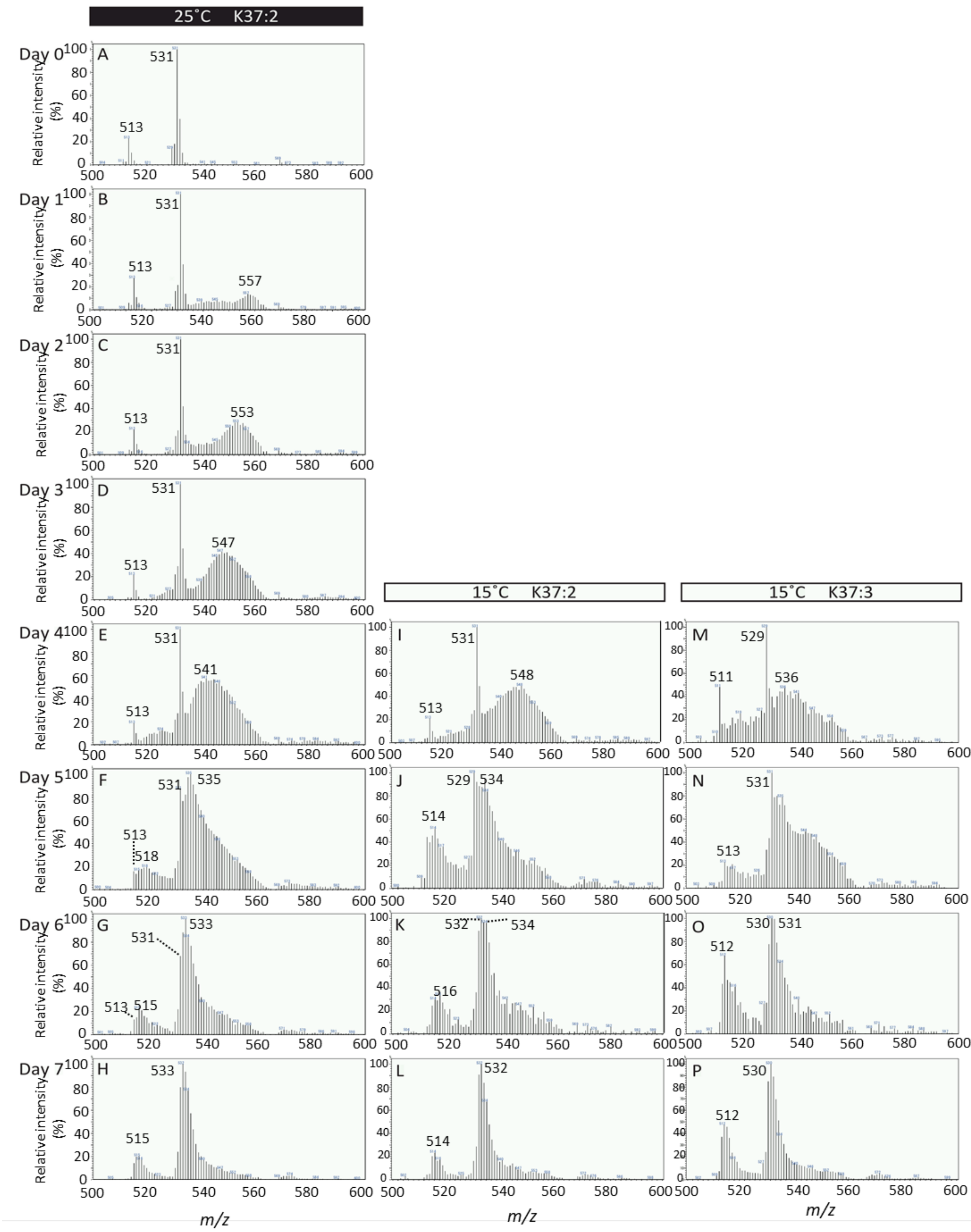

**Figure S2 Changes in mass spectrometric profiles of $C_{38}$ alkenones during the $^{13}$C-labelling experiments of *E. huxleyi* CCMP2090 cells at 25˚C and 15˚C.**

Experiments are same as those in **Fig. 1**. Time course is shown in details with profiles taken every day from Day 0 to 7. Sodium [$^{13}$C]bicarbonate (final concentration, 4 mM) was added on Day 0 for $^{13}$C-labelling experiments. The $^{13}$C-labelling patterns of $C_{38:2}$ alkenones ($K_{38:2}$Me and $K_{38:2}$Et) in cells grown continuously at 25 ˚C are shown in A to H for Days 0 to 7 (left column). The $^{13}$C-labelling patterns of $C_{38:2}$ alkenones ($K_{38:2}$Me and $K_{38:2}$Et) in cells transferred from 25 to 15 ˚C on Day 3 are shown in I to L for Day 4 to 7 (2$^{nd}$-left column). And $^{13}$C-labelling patterns of newly-formed $K_{38:3}$Me and $K_{38:3}$Et at 15 ˚C are shown in M to P for Day 4 to 7 (3$^{rd}$-left column) and in Q to T for Day 4 to 7 (right column), respectively. See Fig. 1 for growth curves and change in alkenone contents. All graphs (**A-T**) represent the spectra of relative intensity of $^{13}$C (%) (y-axis) at each m/z value (x-axis) in mass spectrometric analysis.

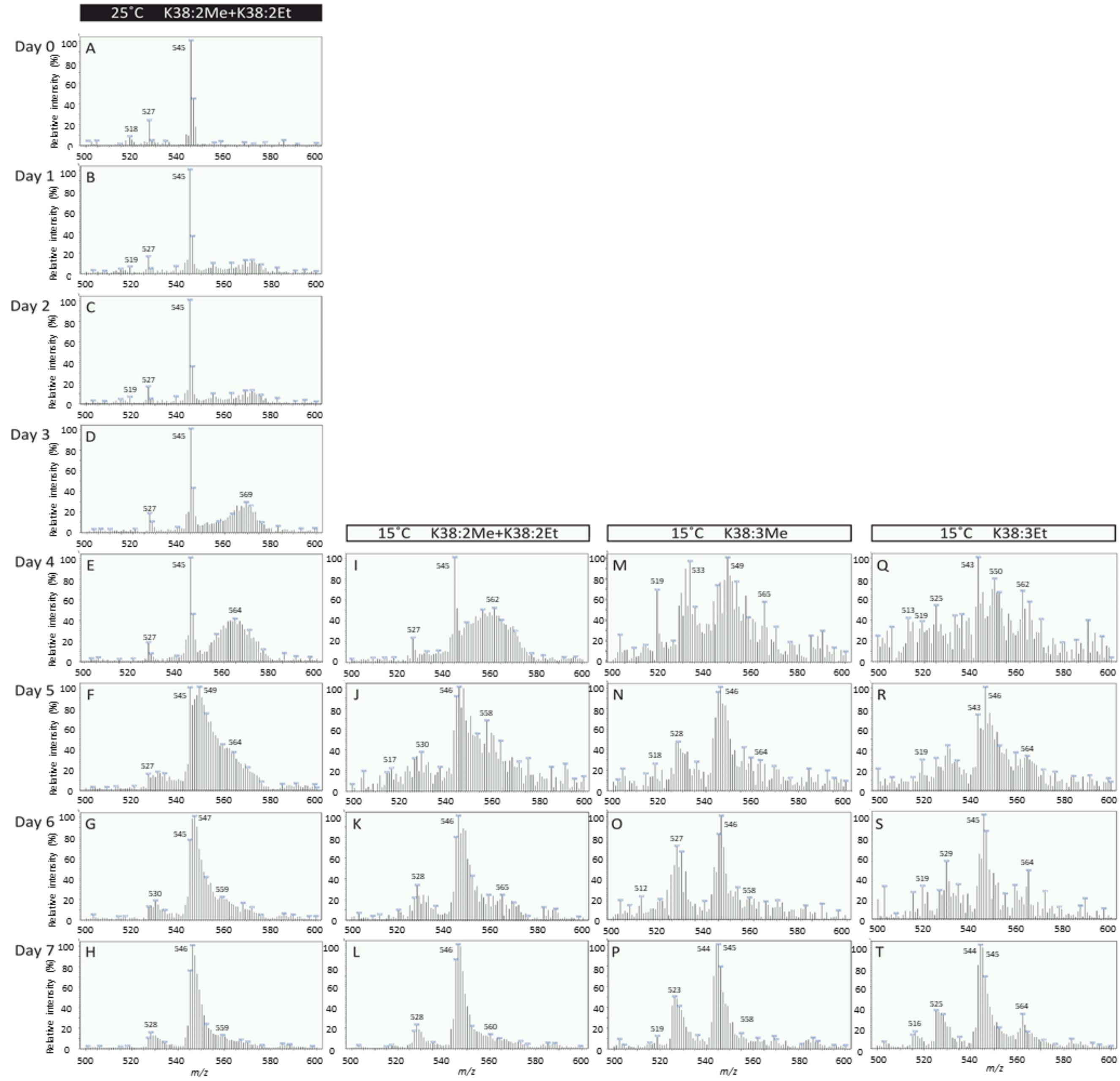

Supplement: Supplementary file 1 — Supplementary Infomation [file 41598_2018_20741_MOESM1_ESM.pdf]
